# Differential expression of immune receptors in two marine sponges upon exposure to microbial-associated molecular patterns

**DOI:** 10.1038/s41598-018-34330-w

**Published:** 2018-10-31

**Authors:** Lucía Pita, Marc P. Hoeppner, Marta Ribes, Ute Hentschel

**Affiliations:** 1 RD3 Marine Microbiology, GEOMAR Helmholtz Centre for Ocean Research Kiel, Kiel, Germany; 2 Institute of Clinical Molecular Biology, Christian-Albrechts University of Kiel, Kiel, Germany; 3 Institute of Marine Science, CSIC, Barcelona, Spain; 4 Christian-Albrechts-University of Kiel (CAU), Kiel, Germany

## Abstract

The innate immune system helps animals to navigate the microbial world. The response to microbes relies on the specific recognition of microbial-associated molecular patterns (MAMPs) by immune receptors. Sponges (phylum Porifera), as early-diverging animals, provide insights into conserved mechanisms for animal-microbe crosstalk. However, experimental data is limited. We adopted an experimental approach followed by RNA-Seq and differential gene expression analysis in order to characterise the sponge immune response. Two Mediterranean species, *Aplysina aerophoba* and *Dysidea avara*, were exposed to a “cocktail” of MAMPs (lipopolysaccharide and peptidoglycan) or to sterile artificial seawater (control) and sampled 1 h, 3 h, and 5 h post-treatment for RNA-Seq. The response involved, first and foremost, a higher number of differentially-expressed genes in *A*. *aerophoba* than *D*. *avara*. Secondly, while both species constitutively express a diverse repertoire of immune receptors, they differed in their expression profiles upon MAMP challenge. The response in *D*. *avara* was mediated by increased expression of two NLR genes, whereas the response in *A*. *aerophoba* involved SRCR and GPCR genes. From the set of annotated genes we infer that both species activated apoptosis in response to MAMPs while in *A*. *aerophoba* phagocytosis was additionally stimulated. Our study assessed for the first time the transcriptomic responses of sponges to MAMPs and revealed conserved and species-specific features of poriferan immunity as well as genes potentially relevant to animal-microbe interactions.

## Introduction

The advent of microbial life on earth predates that of animals by at least 3 billion years^1^. Even today, microorganisms account for most of the life on our planet, both in terms of diversity and biomass^2^. It is therefore not surprising that animals have evolved strategies for interacting with microbes^1,3^. Indeed, all animals engage in stable and highly-specific associations with microbial communities and these symbioses deeply impact animal ecology and evolution^1,3^. The recognition of microbes as evolutionary partners has changed the way we view animal systems and has opened new frontiers of research. A prominent example is the paradigm shift in our understanding of the immune system—from the classical view as conserved defence mechanism against pathogens to the emerging perspective of immunity as rudder that allows the host to navigate the microbial world, mediating both defence and tolerance^4–6^.

A common challenge for all animals is discriminating between microbes in order to maintain a specific microbiome, while also avoiding overgrowth, harmful infections, or energetically-expensive immune reaction to innocuous microbes. Upon microbial encounter, animals detect microbe-derived molecules (microbial-associated molecular patterns, MAMPs), such as lipopolysaccharide (LPS), peptidoglycan, or flagellin, which are absent in eukaryotic organisms^7,8^. Pattern-recognition receptors (PRRs) of the innate immune system recognise these MAMPs and transduce a signal that activates the corresponding immune response^9,10^. Detection of pathogen-derived MAMPs initiates pathogen destruction^11–13^, whereas detection of symbiont-derived MAMPs promotes tolerance^4,7,14,15^. Even in model animals, it is not yet fully understood how the identity of the microorganism shapes the down-stream interpretation of the microbial signal detected by the PRRs. It may be related to specific MAMP structures of certain microbes (e.g.^16,17^,) or to accompanying danger signals in pathogenic infections^18^. In any case, the appropriate response relies on specific recognition and fine-tuned down-stream regulation of the immune response. Due to the absence of an adaptive immune system, three mechanisms have been proposed as molecular basis for specific recognition in invertebrates^19^: (i) high genetic diversity of receptors or immune effectors, (ii) enhanced expression of relevant receptors upon microbial encounter, and (iii) synergistic interactions among immune components.

Several families of animal PRRs are characterized according to the distinct arrangement of conserved protein domains. The Toll-like receptors (TLRs) are membrane-bound receptors with an extracellular domain (leucine-rich repeats in canonical TLRs) that recognizes the MAMPs and an intracellular Toll/interleukin-1 receptor (TIR) domain that triggers a well-characterized signalling cascade. This signalling cascade is present and functional in early-diverging animals^20^. The nucleotide-binding domain and leucine-rich repeat containing receptors (NLRs) are mainly cytosolic receptors that detect signals from microbes, tissue damage, or cellular stress^21^. NLR-mediated activation of the mitogen-activated protein kinase (MAPK) signalling cascade (e.g., p38, JNKs) and caspases results in reactive oxygen species formation, inflammatory processes, production of antimicrobial peptides, as well as cell death^22,23^. Other receptor families, such as the scavenger receptor cysteine-rich (SRCR) and lectins, add to the diverse repertoire of immune receptors found in most animals^10^. Another abundant and diverse class of receptors is the G-protein coupled receptors (GPCRs)^24^. Although they are classically omitted from the PRR group, empirical evidence supports their role in the recognition of microbial signals in both invertebrates and vertebrates^24,25^.

Sponges (phylum Porifera) are among the earliest-diverging multicellular animals and thus considered key to understanding the origins of animal processes, including animal-microbe interactions^26,27^. Due to their sessile filter-feeder lifestyle, sponges constantly encounter microbes from the seawater, which serve as a food source, but at the same time maintain stable species-specific symbiotic communities^28^. The field of sponge symbiosis has consolidated in recent years^29,30^, but it remains largely focused on the microbial side, while host mechanisms for microbial recognition and control are still poorly explored. The genome of *Amphimedon queenslandica* showed, for the first time, the enormous complexity of the Poriferan genomic toolkit^26^. It comprised a high diversity of PRRs^26,31,32^, including expanded NLR and SRCR families^10,32^. Recent genomic and transcriptomic studies in other sponge species confirmed the complex repertoire of PRRs and the presence of key components of immune signalling cascades, such as the TLR-mediated signalling pathway^33–35^. The conserved domain architectures of PRRs, their similarity to vertebrate counterparts, and the striking expansion of PRR families in sponges collectively indicate conserved functions in MAMP recognition and signal transduction^36^. Still, empirical evidence of such functions remains scarce^37–39^.

Here we utilized an experimental approach in order to characterise the suite of PRRs and immune genes involved in the response of sponges to microbial elicitors. We aimed to induce an immune response that would reveal the gene toolkit that is relevant for sponge immunity in the context of microbial recognition. We challenged the sponges *Aplysina aerophoba* and *Dysidea avara* with MAMPs (LPS and peptidoglycan) under controlled conditions in aquaria and assessed their response by way of RNA-Seq analysis. These two Mediterranean sponge species illustrate a long-accepted dichotomy in sponge symbiosis^40^—sponges termed “high microbial abundance” (HMA), like *A*. *aerophoba*, harbour symbiotic communities in densities that are two to four orders of magnitude higher than in the “low microbial abundance” (LMA) sponges such as *D*. *avara*. The HMA-LMA dichotomy involves, in addition to differential symbiont densities, differences in microbial diversity and metabolic features of the sponges^41,42^. Moreover, a recent genomic analysis on HMA and LMA sponge representatives from the Red Sea suggested a more expanded repertoire of immune-related domains in the LMA than the HMA sponges^34^. Previous works reported that sponges can rapidly take up seawater bacteria but are unable to take up their own symbionts, which suggests that sponges are capable of differentiating microbes^43,44^. Therefore, we hypothesise that both sponges rely on differential expression of PRRs and signalling genes to recognize and respond to MAMPs. We also expect species-specific strategies according to their different immune repertoires and HMA-LMA status.

## Methods

### Specimen collection

Specimens of the Mediterranean sponge species *Aplysina aerophoba* and *Dysidea avara* were collected via SCUBA diving at the coast of Girona (Spain) in March 2015 (42.29408 N, 3.28944 E and 42.1145863 N, 3.168486 E; respectively). *A*. *aerophoba* was collected at a depth ca. 3 m and the water temperature at the time of collection was 11 °C. *D*. *avara* was collected at a depth ca. 15 m and the water temperature at the time of collection was 12 °C. Collection was performed in a way that a part of the sponge remained in the substrate, allowing the regeneration of the individual. Sponges were then transported to the Experimental Aquaria Zone (ZAE) located at the Institute of Marine Science (ICM-CSIC) in Barcelona (Spain). Sponges were placed in separated 6 L aquaria in a flow-through system with direct intake of seawater and a circadian cycle of 12 h light/12 h dark using artificial light sources. Sponges were acclimated under these conditions for one week prior to experimentation.

### MAMP challenge

The same experimental design was applied to each sponge species and experiments were conducted consecutively. Before the experiments, sponges were kept overnight in 1µm-filtered seawater and an additional 0.1 µm-filter was applied for 3 h before the experiments. The flow-through was stopped during the experiment and small aquarium pumps were applied to ensure mixing of the water in the aquarium. Sponges were randomly assigned to each treatment (n = 5 individuals per treatment). In the MAMP treatment, sponges were injected with LPS (source: *Escherichia coli* O55:B5, Sigma L2880) and peptidoglycan (source: *Staphylococcus aureus*, Sigma 77140) (500 µL of a final concentration 1 mg/mL in sterile artificial seawater, 1:1), with the aim of triggering an acute immune response. Sponges in control treatment were injected with sterile artificial seawater (500 µL). Treatments were directly injected into the tissue at 3–5 different spots. Sponge pumping activity was assessed visually (i.e., open oscula). For each individual, one tissue sample from one of the injection sites was collected at 1 h, 3 h and 5 h post-injection. Samples were placed in RNAlater, maintained overnight at 4 °C, and stored at −80 °C until processed. For further analysis, 3 samples per time point and treatment were randomly selected.

### Extraction and sequencing of eukaryotic mRNA

Eukaryotic mRNA was obtained following the protocol described by Moitinho-Silva *et al*.^45^. Briefly, cells were mechanically lysed and total RNA was extracted using the AllPrep DNA/RNA kit (Qiagen, Germany). Contaminating genomic DNA was removed using the RQ1 RNase-free DNase (Promega, USA). RNA quantity and integrity were analyzed using Invitrogen^TM^ Qubit^TM^ fluorometer and Experion System (Bio-Rad, USA). Sponge mRNA was isolated from ca. 100 µg of total RNA (obtained from pooling 6–10 extractions from the same biological replicate) using a Poly(A) Purist MAG kit (Ambion, USA) with two round of poly(A) purification. Library preparation (including the reverse transcription of the mRNA into cDNA) and sequencing was performed at the IKMB Kiel (Germany). The cDNA libraries were prepared using the Illumina TruSeq stranded mRNA kit and paired-end sequenced on the HiSeq. 2500 platform using HiSeq v4 reagent kit (Illumina, Inc., USA).

### Data filtering, *de novo* transcriptome assembly and functional annotation

Given the lack of reference genomes for these sponges, a reference transcriptome was assembled *de novo* for each species. Raw Illumina reads were filtered to remove adapters and low-quality reads in Trimmommatic-version 0.35^46^ (filtering parameters- LEADING:3 TRAILING:3 SLIDINGWINDOW:4:15 MINLEN:75). Read quality was visualised in FastQC. Additional filtering of prokaryotic and microbial eukaryotic reads was performed in the classifier Kaiju^47^, in greedy-5 mode (version and database accessed in October 2016). The remaining reads of samples belonging to the same species were combined to create *de novo* reference assemblies in Trinity-version 2.2.0^48^, following the general pipeline for stranded libraries. Statistics from the assemblies were obtained in Trinity and TransRate-version 1.0.2^49^. Completeness was assessed by comparing the assemblies against the Metazoa reference data in BUSCO-version 1.22^50^, trans mode). Assemblies were annotated in Trinotate-version 3.0.1 (e-values < 1 e^−5^), a comprehensive suite that includes homology search to publicly available data (BLAST+/SwissProt), protein domain identification (HMMER/Pfam), protein signal peptide and transmembrane domain prediction (signalP/tmHMM), as well as eggNOG, GO and KEGG annotation. Those contigs with blast matches to Bacteria, Archaea, or Virus were further removed from the reference assembly. The annotation report was manually screened for the presence of the most common PRR families based on the PFAM annotation. Specifically, non-canonical TLR were identified by the presence of the TIR domain (PF01582), in combination with Ig-like domains (PF00047), NLRs by the presence of the NACHT domain (PF05729), in combination with leucine-rich repeat (LRR) domains (PF13516), and SRCRs by the presence of the SRCR domain (PF00530 or PF15494).

### Transcript quantification and differential gene expression analysis

Following the Trinity pipeline, gene abundance was estimated separately for each sample by RSEM bowtie2-based quantification (version 1.2.19). Trinity outputs include the estimates for genes (Trinity components) and isoforms (Trinity transcripts). Distinguishing true isoforms from chimeras or fragmented genes remains a challenge; thus, the analysis presented here is based on gene (Trinity component) abundances. Differential gene expression analysis within each time point (i.e. 1 h, 3 h, and 5 h) was performed in edgeR (exact test mode) as implemented in the Trinity pipeline (default parameters). Differentially expressed genes (DEGs) in the MAMP compared to control treatment were defined by False Discovery Rate –corrected (FDR) p-value < 0.005 and log_2_|fold change| ≥ 2. For comparison, DeSeq 2 tool (as implemented in Trinity pipeline) was also tested for identification of DEGs in order to check for consistency with edgeR results. DESeq 2 found a higher number of DEGs than edgeR for the same significance threshold (Supplementary Fig. S3). Importantly, 91% and 100% of edgeR-DEGs (FDR p-value < 0.005) were consistently retrieved by DESeq 2 in *A*. *aerophoba* and *D*. *avara*, respectively. Therefore, we further explored and report here the edgeR-based results.

For a DEG annotated as a *GPCR* in *A*. *aerophoba*, we confirmed its presence in other sponge species by performing a blast search (at protein level, 1e^−5^ threshold) against a custom local database constructed from publically available transcriptomic information for 17 sponge species (*Amphimedon queenslandica*, *Ephydatia muelleri*, *Haliclona amboinensis*, *H*. *tubifera*, *Leucosolenia complicata*, *Oscarella carmela*, *Oscarella* sp., *Stylissa carteri*, *Sycon ciliatum*, *Xestospongia testudinaria*, *Chondrilla nucula*, *Corticium candelabrum*, *Ircinia fasciculata*, *Petrosia ficiformis*, *Pseudospongosorites suberitoides*, *Aphrocallistes vastus*, and *Sycon coactum*). We also searched for similar genes (blast search at protein level, e-value < 1e^−5^) against other marine invertebrates available in the Ensembl Metazoa database (i.e., *Mnemiopsis leidyi*, *Nematostella vectensis*, *Strongylocentrotus purpuratus*) and against vertebrate species available in the Ensembl database (i.e., *Homo sapiens*, *Danio rerio*, and *Xenopus tropicalis*). The protein alignment was built in MAFFT version 7.402 as implemented in CIPRES Science Gateway, with E-INS-i strategy and default parameters, and further visualized in Jalview Desktop^51^. The resulting alignment was used for phylogenetic tree construction in RAxML version 8.2.10^52^ within CIPRES Science Gateway, with 500 rapid bootstrap inferences and maximum likelihood search under GAMMA and WAG substitution model. The phylogenetic tree was annotated in FigTree v1.4.3 (http://tree.bio.ed.ac.uk/software/figtree/).

The set of DEGs when applying a more relaxed significance threshold, FDR p-value < 0.05, was explored via interaction network analysis in STRING-version 10.5^53^, accessed in October 2017. We used the protein name of the top blast hit (HUGO nomenclature) of Trinotate annotation as input for STRING. STRING searches for the corresponding COG annotations and depicts a network of COG-COG interactions based on multiple types of evidences (e.g. known interactions from curated databases and experiments or predicted interactions based on gene co-occurrence and gene neighbourhood)^53^. We applied a minimum interaction score of 0.700 (high confidence). For *A*. *aerophoba*, two networks were created: one for the set of up-regulated genes, the other for the down-regulated genes. For *D*. *avara*, the number of annotated genes was relatively low, and therefore, a single network combining both up-regulated and down-regulated genes was created.

## Results

### Sequencing and *de novo* transcriptome assemblies

The number of paired-end Illumina reads generated in this study is summarised in Table 1. They originated from a total of 18 samples from *A*. *aerophoba* and 17 samples from *D*. *avara*, corresponding to three biological replicates per treatment within each of the three time points (except for *D*. *avara* 1 h post-MAMP treatment, for which the library construction of one replicate failed). The surviving paired reads post-filtering (Table 1) were used for generating a *de novo* reference assembly for each species. The statistics of the resulting reference transcriptomes are summarised in Table 2. Those contigs with similarity (blast hits) with Bacteria, Archaea, or Virus-derived sequences were removed from the reference assembly (Table 2, filtering after annotation). BUSCO assessments revealed that 69% and 70% of the 843 core Metazoan genes were detected in *A*. *aerophoba* and *D*.*avara* reference assemblies, respectively, with 21% of the genes found as fragments.

**Table 1:** Number of read pairs (million reads).

	*A. aerophoba* raw	*A. aerophoba* clean	*A. aerophoba* eukaryote	*D. avara *raw	*D. avara* clean	*D. avara* eukaryote
average per library (±standard error)	20.8 ± 2.2	17.9 ± 2.1	13.3 ± 1.6	18.4 ± 1.4	14.2 ± 1.0	10.3 ± 0.7
total	374.2	320.9	239.4	341.2	264.7	176.1

“Raw” refers to the output from sequencing; “clean” to surviving pairs after trimming in trimmomatic-v0.35; and “eukaryote” to pairs identified as non-prokaryotic and non-microbial eukaryote by kaiju^47^ (see methods).

**Table 2:** Statistics of the *de novo* transcriptomic assemblies.

Statistics	*A. aerophoba*	*D. avara*
Transcripts -Trinity isoforms (transcripts > = 300 bp)	638913 (324604)	740537 (489719)
Genes-Trinity components (genes > = 300 bp)	505816 (227119)	592747 (362170)
Average transcript length, nucleotides	578	698
Transcripts with open reading frames (%)	553378 (86.6)	424901 (57.4)
Non-redundant eukaryotic protein-coding genes	26736	47936
N50 (considering only transcripts > = 300 bp)	500 (736)	669 (835)
Total assembled bases, Mb	292.6	413.9
**Filtering after Blast search:**		
-Transcripts (transcripts > = 300 bp)	618508 (310083)	734795 (484868)
-Genes (genes > 300 bp)	480475 (217086)	578071 (358874)

Transcripts refers to Trinity isoforms, genes refers to Trinity components.

Bp: base pair.

### Diverse repertoire of putative PRRs in reference transcriptomic assemblies

Based on the presence of conserved domains (Pfam annotation), we identified putative PRRs within the families of non-canonical TLRs, NLRs, and SRCRs in the reference transcriptomes of *A*. *aerophoba* and *D*. *avara*. *Bona fide* NLRs are characterised by the presence of NACHT and leucine-rich repeat (LRR) domains (as in Yuen *et al*.^32^). In the *A*. *aerophoba* reference transcriptome, only one gene (Trinity component *TR172818_c2_g1*) showed this architecture across a complete open reading frame (Supplementary Table S1). However, 75 additional genes contained a NACHT domain and could potentially belong to the NLR family but lacked the LRR domain (Supplementary Table S1). In *D*. *avara*, 80 *bona fide NLR* genes were detected of which 39 were complete (Supplementary Table S2). The number of additional NACHT domain-containing genes in the *D*. *avara* reference transcriptome extended to 390 (Supplementary Table S2). The reference transcriptomes of *A*. *aerophoba* and *D*. *avara* also included >250 genes containing single or multiple SRCR domains, sometimes in combination with other conserved domains such as fibronectin III, protein kinases, Sushi repeats, or epidermal growth factor-like domains (Supplementary Tables S1, S2). While sponges lack *bona fide* TLR, they do contain Immunoglobulin-TIR receptors characterised by an intracellular TIR domain (which is homologous to the TIR domain in TLR in Eumetazoan^54^) but with immunoglobulins instead of LRRs as extracellular domain^31^. We detected a single gene in *A*. *aerophoba* (*TR170373_c0_g1*, Supplementary Table S1) and two genes in *D*. *avara* (*TR163581_c0_g2* and *TR169736_c5_g2*, Supplementary Table S2) presenting this architecture. In addition, KEGG annotation identified components of the TLR signalling pathway (Supplementary Figs S1, S2), as reported in other sponge species^35,54^.

### Transcriptomic profiles in response to MAMPs

**Figure 1:**
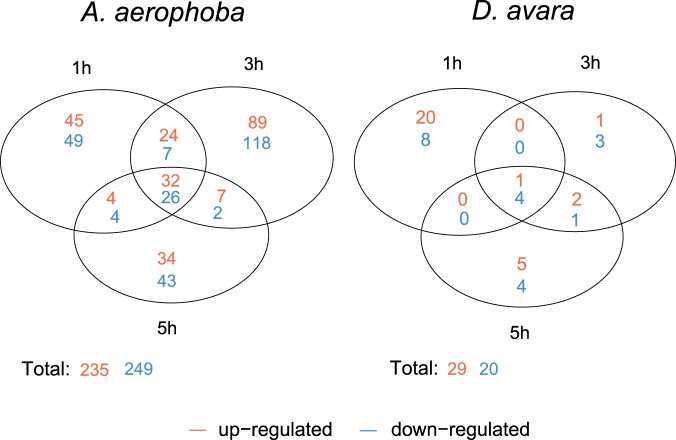
Numbers of DEGs those were either common or specific for each time point (1 h, 3 h, 5 h) in each sponge species upon MAMP treatment. Within each time point, DEGs were identified by comparing gene expression levels in MAMP relative to control treatment and according to the defined threshold FDR p-value < 0.005 and log2|FC| ≥ 2 expression, as calculated in edgeR. “Up-regulated” and “down-regulated” refers to genes with higher and lower expression in MAMP than in control treatment, respectively.

**Figure 2:**
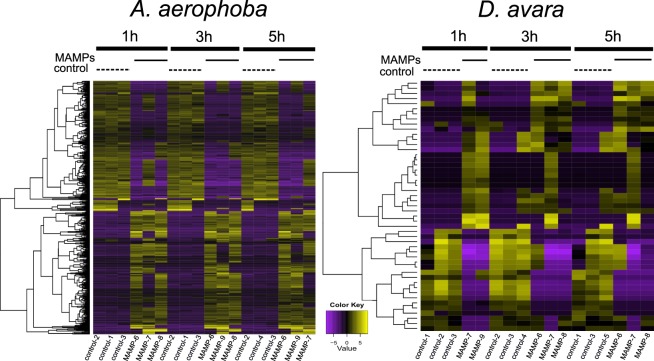
Heatmaps of differentially expressed genes upon MAMP treatment. Heatmaps show relative expression levels of each DEG (rows, hierarchically clustered) in each sample (columns) from *A*. *aerophoba* (left) and *D*. *avara* (right). DEGs are defined by FDR p-value < 0.005 and log2|FC| ≥ 2 expression (4-fold change), as calculated in edgeR. Expression values are log2-transformed median-centred TMM-normalised values.

Overall, 83.35 ± 0.21% and 82.17 ± 0.26% of the reads in the samples aligned to the corresponding transcriptome reference in *A*. *aerophoba* and *D*. *avara*, respectively (average ± standard error). Next, gene expression levels in MAMP challenge treatment were compared to those in the control treatment at each time point (1 h, 3 h, and 5 h). DEGs were defined by log_2_|FC| ≥2 (4-fold change) and FDR p-value < 0.005. The DEGs were classified as up-regulated or down-regulated in the MAMP treatment when compared to expression levels in the control treatment. Overall, a higher number of DEGs was detected in *A*. *aerophoba* than in *D*. *avara* (Fig. 1). A total of 235 and 249 genes were identified as up-regulated and down-regulated, respectively, in *A*. *aerophoba*. In *D*. *avara*, the total number of DEGs was 29 up-regulated and 20 down-regulated. Most DEGs detected within a sponge species were time-specific (Fig. 1). In *A*. *aerophoba*, the highest number of DEGs was detected 3 h after MAMP challenge. In *D*. *avara*, the highest differential expression occurred 1 h after treatment; but only 2 replicates from the MAMP treatment were available for this time point, which could have influenced the observed trend. Heatmaps illustrate the consistency of DEG-expression profiles among biological replicates in each treatment and time point (Fig. 2). The full results from the differential expression analysis in edgeR are reported in Supplementary Tables S3 and S4 and the full annotation report for DEGs is available as Supplementary Tables S6 and S7.

### PRR expression and signalling in response to MAMPs (FDR p-value < 0.005)

**Figure 3:**
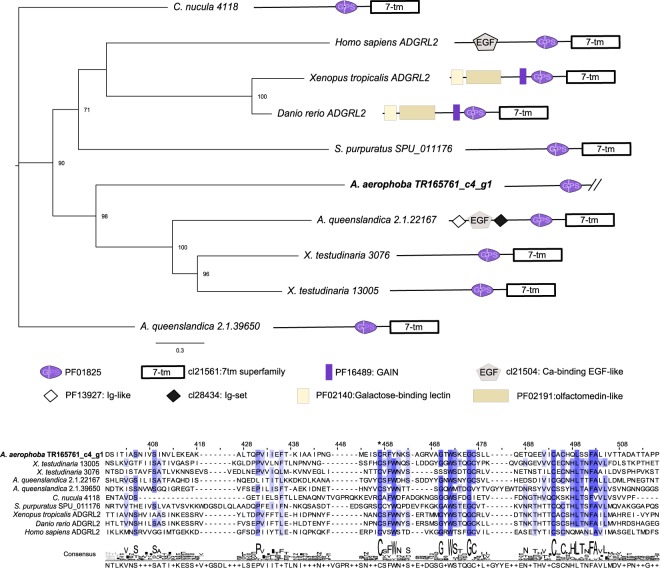
Phylogenetic analysis of the *A*. *aerophoba GPCR* up-regulated gene *TR165761_c4_g1*. A part of the alignment is reported. The star (*) denotes the beginning of the GPS motif. Conserved residues (identical in all sequences) are shown in dark blue, and those identical in at least 50% of the sequences are in light blue. A schematic representation of the domain architecture of each gene is provided. As *TR165761_c4_g1* gene is incomplete, we removed the 7tm domain from the other protein sequences included in the alignment prior to tree construction by maximum likelihood analysis. Node labels represent bootstrap support greater than 50% of 500 pseudoreplicates.

Based on Pfam domain architectures, several putative PRRs were identified as differentially expressed in response to the MAMP challenge (Table 3). In *A*. *aerophoba*, the repertoire of receptors that were differentially expressed included one gene with a SRCR domain (*TR13528_c0_g1*, partial gene). We also include in this category a gene identified as a *GPCR* by the presence of a GPS motif (PF01825: GPCR proteolytic site). Further phylogenetic analysis of this gene suggests that it belongs to the group of adhesion GPCRs, with similarity to the vertebrate group I (*ADGRL2* genes, also known as *latrophilin-2*) (Fig. 3). In *D*. *avara*, *bona fide NLR*s were significantly up-regulated upon MAMP challenge (Table 3). Within them, the *TR172577_c0_g1* gene was among the 10 highest differentially expressed genes at each time point (in terms of fold change and FDR p-value) and contained a predicted transmembrane domain (Supplementary Table S6). Also, a leucine-rich repeat-containing gene and several genes containing fibrinogen-related domains were differentially expressed and included as putative PRRs (Table 3). The fibrinogen domain containing genes showed similarity to vertebrate ficolins and angiopoietin-related genes (blastp, e-value < 1e^−5^). Fibrinogen-like proteins have been proposed as potential immune receptors in molluscs and other invertebrates^55^. Potential receptors according to sequence similarity, but without the corresponding conserved domains, are included in Tables 4, 5 and Supplementary Table S5.

**Table 3:** Differential expressed genes identified as immune receptors in A. aerophoba and D. avara, according to the presence of conserved domains.

	Description	Domain architecture	GeneID	Time	Log FC	FDR
***A.aerophoba***	SRCR		*TR13528_c0_g1*	1h; 3h; 5h	8.6; 8.8; 9.3	0.035; 0.020; **2.1 e-5**
	G-protein coupled receptor		*TR165761_c4_g1*	1h	11.9	**0.003**
***D.avara***	*Bona fide* NLR		*TR146630_c0_g1*	1h	10.0	**6.8 e-4**
			*TR172577_c0_g1*	1h; 3h; 5h	10.1; 11.0; 9.6	**4.4 e-4;** **0.001;** **0.001**
	LRR-containing gene		*TR126682_c0_g3*	5h	−10.2	**2.5 e-4**
	Fibrinogen-like genes		*TR136253_c0_g1*	1h	11.2	**4.3 e-4**
			*TR164124_c0_g1*	1h	11.2	**4.3 e-4**
			*TR286444_c0_g1*	1h; 3h; 5h	−15.0; −15.4; −13.6	**5.4 e-9;** **6.7 e-8;** **2.4 e-7**
			*TR83489_c0_g1*	1h; 3h; 5h	−12.2; −12.6; −11.3	**9.3 e-4;** **6.8 e-7;** **4.3 e-6**
			*TR261782_c0_g1*	1h; 3h; 5h	−13.0; −13.6; −11.1	**4.3 e-4;** **1.6 e-6;** **1.1 e-4**

SRCR domain (PF00530),  ATPase family associated with various cellular activities{PFD0004),  NACHT domain (PF05729),  GPCR proteolysis site.GPS.motif (PF01825),  DEATH domain (PF00531)  Fibrinogen_C. Fibrinogen bele and gamme chains. C-terminal globular domain (PF00147)  Leucine rich repeat. LRR_6. domain (PF13516). Genes with FDR p-value < 0.005 at least at one time point. FDR p-values < 0.005 are highlighted in bold. For the other time points, only FDR p-values <0.05 are shown. Log FC: log_2_ (fold change). Positive values of Log FC denote up-regu lated genes; negative values of log FC denote down-regulated genes. FDR: false discovery rate-corrected p-value.

**Table 4:** Differentially expressed genes (FDR p-value < 0.005) *in A. aerophoba*.

Gene Description	Gene IDs	Time	LogFC	FDR
**Recognition/ adhesion/extracellular matrix**				
Ankyrin repeats-containing gene	*TR175111_c5_g9*	1h; 3h; 5h	*4.6;* *6.9;* *6.2*	**1.6 e-4;** **1.1 e-6;** **7.8 e-7**
	*TR171083_c2_g19*	3h	*4.4*	**6.3e-5**
Sushi-domain containing gene	*TR171108_c0_g5*	3h	*8.2*	**3.8 e-10**
Matrilin-2 like (Calcium-binding EGF-like, Sushi and Ig-like domain containing gene)	*TR145455_c0_g2*	1h; 3h	*11.7;* *11.8*	**0.004** **0.002**
C-type lectin family	*TR171108_c0_g16*	3h	*8.2*	**9.2 e-5**
Tetrapeptide repeat-containing gene	*TR166645_c4_g19*	3h	*7.2*	**1.8 e-6**
SAM-domain containing gene	*TR173732_c1_g2*	3h	*5.4*	**0.001**
FnIII domain-containing gene	*TR171190_c4_g1*	1h; 3h; 5h	*9.7;* *9.9;* *10.4*	**2.0 e-7;** **3.7 e-9;** **5.7 e-9**
	*TR168661_c2_g1*	1h	*5.9*	**1.5 e-4**
	*TR176105_c52_g28*	1h	*4.5*	**0.001**
	*TR170262_c4_g21*	3h	−8.1	**2.7 e-4**
	*TR170248_c3_g2*	5h	−11.5	**4.8 e-3**
Hemicentin-like TM signalling peptide	*TR172325_c2_g1*	3h	*9.5*	**9.7 e-4**
	*TR171647_c5_g11*	3h; 5h	*11.9;* *13.7*	**0.003;** **0.002**
Folate receptor	*TR173479_c1_g6*	1h; 3h; 5h;	*6.3;* *7.8;* *7.8*	**5 e-4;** **1.7 e-4;** **4.6 e-3**
Immunoglobulin superfamily	*TR169220_c5_g12*	3h	−4.5	**6.6 e-5**
Galectin	*TR246625_c0_g1*	3h	−9.5	**0.002**
FnIII domain and Sushi repeat-containing gene	*TR167502_c4_g9*	5h	−4.6	**0.001**
GPCR	*TR175974_c14_g10*	3h	−5.3	**0.002**
Collagen	*TR174460_c0_g11*	1h	−7.1	**0.004**
	*TR170657_c2_g1*	3h	−4.4	**1.3 e-5**
	*TR156245_c1_g3*	1h; 3h	*7.9;* *7.5*	**0.002;** **4.7 e-3**
Von Willebrand factor type A domain-containing gene	*TR172723_c2_g1*	3h	*5.1*	**0.002**
	*TR118838_c1_g1*	3h; 5h	*14.0;* *12.7*	**0.004;** **2.0 e-8**
	*TR170575_c0_g1*	1h	−6.3	**0.004**
LIM and SH3 like	*TR167199_c6_g3*	5h	−7.4	**4.9 e-3**
Myosin light chain	*TR172325_c2_g1*	3h	*9.5*	**9.7 e-4**
Coadhesin-like	*TR172756_c2_g3*	5h	−3.8	**0.003**
Dynein	*TR169274_c2_g8*	5h	*8.8*	**1.3 e-4**
**Chaperones**				
Heat shock protein	*TR169461_c3_g6*	1h; 3h	*5.6;* *8.0*	**0.001;** **1.7 e-6**
**Signalling cascades**				
Dynamin family	*TR167095_c0_g2*	3h	*4.3*	**0.002**
	*TR165470_c0_g1*	1h; 3h; 5h	*8.2;* *7.9;* *10.1*	**1.4 e-4;** **1.3 e-4;** **5.2 e-7**
	*TR162616_c0_g2*	3h	−7.2	**0.002**
DEATH domain-containing gene	*TR174492_c12_g1*	1h; 3h; 5h	*11.5;* *9.3;* *12*	**1.0 e-8;** **7.9 e-8;** **1.3 e-8**
Transmembrane protein 87B like	*TR58530_c0_g1*	3h	*7.9*	**7.2 e-4**
Ras family	*TR136365_c0_g2*	1h	−7.9	**3.2 e-4**
Tyrosine phosphatase	*TR121398_c1_g1*	5h	-3.9	**4.9 e-3**
Serine Threonine protein kinases	*TR173438_c1_g1*	3h	−9.8	**1.5 e-10**
	*TR177584_c0_g1*	3h	−10.5	**5.8 e-12**
	*TR172256_c3_g1*	1h	−6.0	**8.0 e-4**
Tetraspanin	*TR173370_c7_g19*	1h	−7.7	**7.3 e-4**
Calx-beta domain containing gene	*TR175997_c37_g26*	3h	−4.9	**1.6 e-4**
	*TR166176_c1_g2*	3h	−8.1	**9.2 e-5**
**Transport**				
Calcium-binding protein like	*TR175869_c15_g1*	1h	*5.2*	**0.002**
Sodium/Calcium exchanger	*TR151061_c4_g2*	3h	−6.3	**7.5 e-4**
**Apoptosis**				
CARD-domain containing gene	*TR173078_c1_g5*	1h; 3h; 5h	*5.7;* *4.5;* *5.1*	**4.9 e-4;** **2.3 e-4;** **2.5 e-4**
Pro-apoptotic serine protease–like TM signalling peptide	*TR162574_c0_g1*	1h	*9.8*	**0.003**
Tax1-binding protein	*TR173370_c5_g2*	1h	*5.1*	**2.5 e-4;**
**Ubiquitination**				
Ubiquitin ligase	*TR163420_c1_g4*	1h	*11.2*	**8.4 e-11**
	*TR47283_c0_g1*	5h	−8.2	**6.4 e-4**
	*TR175961_c0_g1*	3h	−12.8	**7.7 e-8**
F-box like	*TR165962_c2_g1*	1h	−5.8	**0.003**
Kelch motif containing gene	*TR173192_c3_g1*	1h	−5.7	**1.0 e-4**
**Others**				
DD3-3	*TR138068_c0_g1*	3h	*7.2*	**2.0 e-6**
Defence protein 3-like	*TR85826_c1_g3* *TR85826_c1_g2*	3h 5h	*7.5* −8.7	**0.002** **0.002**

Gene description based on domain annotation and/or blast results (e-value < 1e^−5^). Supplementary Information provides full information on annotation (including e-values) (Supplementary Table S6) and full DGE results (Supplementary Table S3), here we provide rounded log_2_ fold change and FDR p-values. Log FC: log_2_ (fold change). Positive values of Log FC denote up-regulated genes and are in italic; negative values of log FC denote down-regulated genes and are in underline. FDR: false discovery rate-corrected p-value. EGF: epidermal growth factor; Ig: immunoglobulin; fnII: fibronectin III; TM: transmembrane; GPCR: G-protein coupled receptor.

**Table 5:** Annotated DEGs (FDR p-value < 0.005) in *D*. *avara*.

Gene Description	Gene IDs	Time	LogFC	FDR
**Recognition/cell adhesion/protein binding**				
Fibrinogen	*TR120914_c5_g8*	1 h	*11.2*	**8.3 e-5**
Ig superfamily	*TR137811_c2_g1*	5 h	*10.0*	**0.001**
Ank repeats and ion transport domain-containing gene	*TR142305_c0_g2*	1 h	*9.2*	**0.004**
Gene containing Ig domains and a CARD domain	*TR154561_c0_g1*	1 h	*10.5*	**4.3 e-4**
**Signalling cascade**				
DEATH domain-containing gene (CRADD-like)	*TR165768_c5_g2*	1 h	*11.2*	**8.3 e-5**
Serine/Threonine protein receptor-like kinase	*TR23945_c0_g1*	1 h	*10.2*	**4.5 e-4**
TRAF2	*TR153933_c4_g2*	1 h	*9.8*	**8.3 e-5**
Kelch motif containing gene	*TR146020_c0_g1*	3 h; 5 h	−12.6; **−** 13.5	**4.6 e-5;** **2.4 e-7**
**Lipid-mediated signalling**				
Phospholipase D	*TR123257_c3_g1*	1 h	*9.6*	**0.002**
**Extracellular matrix**				
Collagen	*TR287787_c0_g1*	1 h; 3 h; 5 h	−11.0; −12.1; −11.6	**0.003;** **1.4 e-4;** **1.1 e-4**
**Chaperone**				
Heat shock protein 70	*TR98706_c0_g1*	3 h	*12.2*	**2.6 e-4**
**DNA regulation**				
Histone	*TR1159_c0_g1*	3 h	−7.1	**0.004**

Gene description is based on domain annotation and/or blast results. Supplementary Information provides full information on annotation (including e-values) (Supplementary Table S7) and full DGE results (here we provide rounded log_2_ fold change and FDR p-values, full values are reported in Supplementary Table S4). Log FC: log_2_ (fold change). Positive values of Log FC denote up-regulated genes and are coloured in orange; negative values of log FC denote down-regulated genes and are coloured in blue. FDR: false discovery rate-corrected p-value. Ig: immunoglobulin; Ank: Ankyrin; CARD: caspase recruitment domain.

Genes involved in signal transduction (e.g., kinases), chaperones (i.e., *hsp70*), and genes related to adhesion and extracellular matrix were differentially expressed upon MAMP challenge in both species (Tables 4 and 5). We also detected differential expression of genes related to ubiquitination (i.e., ubiquitin ligases) and apoptosis (Tables 4 and 5). In *A*. *aerophoba* (Table 4), the set of DEGs included genes with conserved domains such as ankyrin repeats, immunoglobulin domains, Sushi and fibronectin III domains or tetrapeptide repeats that could be involved in recognition, adhesion, and cell-cell interactions. The *A*. *aerophoba* gene *TR175974_c14_g10*, which was identified as a GPCR by sequence similarity but not by Pfam domain architecture, was therefore excluded from Table 3 and included in Table 4. According to blast results, several genes potentially involved in GPCR signalling were also significantly differentially expressed upon treatment in this sponge (Table 4). Signalling transduction in *A*. *aerophoba* was further mediated by a DEATH -domain containing gene as well as by several mitogen-activated protein kinase kinase kinases (MAPKKK), which were all down-regulated (Table 4). In *D*. *avara*, the genes involved in recognition, adhesion and cell-cell communication were all up-regulated (Table 5). Signalling transduction was mediated by protein kinases and serine/threonine protein kinases, which were up-regulated too (Table 5). DEGs related with apoptosis were up-regulated 1 h post-treatment in *D*. *avara*. And this sponge up-regulated a gene annotated as *phospholipase D*, which may be involved in lipid metabolism and in the phosphatidylinositol signalling pathway.

DEGs in *A*. *aerophoba* included genes with functions in metabolic processes (Table S5), such as lipid metabolism (e.g., long-chain-fatty-acid-CoA ligases). Other functions under regulation in this species were chromatin remodelling and transcription (e.g., differential expression of DNA-binding proteins and transcription factors) (Table S5). Also, a gene with similarity to *Dictyostelium discoideum DD3-3* gene (*DDB_G0283095*) was up-regulated 3 h after treatment (Table 4). Homologs of this gene are present in other invertebrates, including cnidarians and echinoderms, but are absent in Vertebrata. In *D*. *discoideum*, a *DD3-3* knockout yields faster cell aggregation than in the wild type and compromised cAMP signalling pathway^56^. Another DEG in *A*. *aerophoba* contained a Reeler domain (PF02014), similar to insect defence proteins (Table 3), which may have antimicrobial activity. Several genes remained unidentified due to a lack of similarity with genes in public databases or conserved domains. For example, in *A*. *aerophoba*, the gene *TR170260_c3_g2* was within the top DEGs at all time points (in terms of fold change and FDR p-value) and was identified as a non-transmembrane signalling peptide but no further annotation was available for this gene. Several DEGs within *D*. *avara* which lack annotation were identified as signalling peptides (Supplementary Table S7).

### COG network analysis (FDR p-value < 0.05)

**Figure 4:**
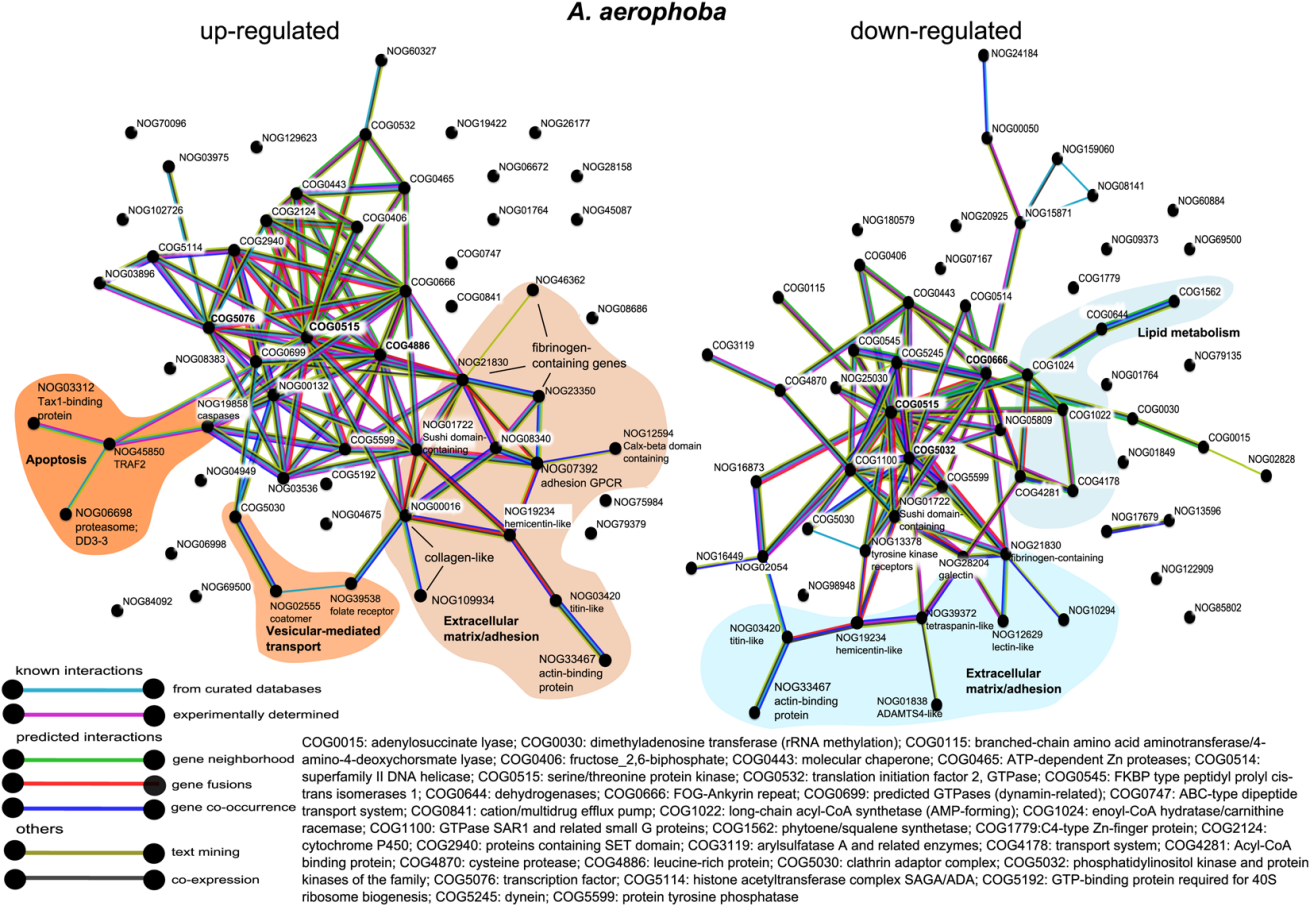
COG association network analysis from annotated differentially expressed genes in *A*. *aerophoba* upon MAMP treatment. Networks of up-regulated and down-regulated DEGs (FDR p-value < 0.05; log2|FC| ≥ 2), as obtained in STRING. The reference protein names identified in Trinotate were used as input. STRING searches for COG annotations and calculates and depicts the association network. Edges represent protein-protein associations coded by colour according to the type of evidence for the shown interaction (see legend). Minimum required interaction score: 0.700 (high confidence). NOG means “non-categorised orthologous group”.

**Figure 5:**
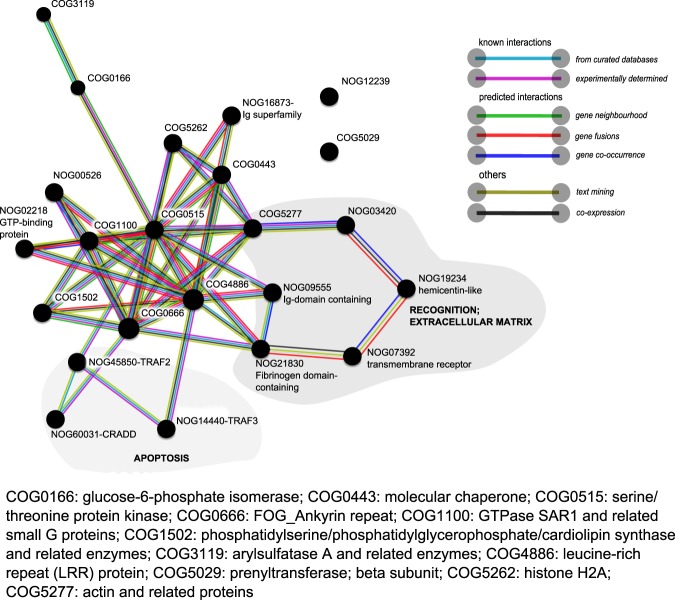
COG association network analysis from annotated differentially expressed genes in *D*. *avara* upon MAMP treatment. Network of annotated DEGs (FDR p-value < 0.05; log2|FC| ≥ 2), as obtained in STRING. The reference protein names identified in Trinotate were used as input. STRING searches for COG annotations and calculates and depicts the association network. Edges represent protein-protein associations coded by colour according to the type of evidence for the shown interaction (see legend). Minimum required interaction score: 0.700 (high confidence). The network includes both down-regulated and up-regulated genes. NOG means “non-categorised orthologous group”.

We also explored the set of DEGs when a more relaxed significance threshold was applied (FDR p-value < 0.05; annotation in Supplementary Tables S6 and S7) to probe for further support of the biological processes activated upon MAMP treatment. In both species, the complex network represented a signalling cascade mediated by kinases (Figs. 4 and 5). In *A*. *aerophoba*, the groups of serine-threonine protein kinases (COG0515) and the ankyrin repeat-containing genes (COG0666) occurred in multiple interactions in both the up-regulated and the down-regulated networks (Fig. 4). In the network of up-regulated genes (Fig. 4, left side), the central nodes (in terms of number of interactions) were leucine-rich repeat proteins (COG4886) and transcription factors involved in chromatin remodelling (COG5076). In the network of down-regulated genes (Fig. 4, right side), the category of phosphatidynositol-3 (PI-3) kinases (COG5032) was also a central node and it connected with other kinases as well as with a network of genes related with lipid metabolism (COG1022; COG1024; COG1562; COG4281). In *D*. *avara*, up-regulated and down-regulated genes were analysed in a single network (Fig. 5). Serine-threonine kinases (COG0515), as well as the category of leucine-rich repeat proteins (COG4886) were the COGs with the highest number of connections (Fig. 5). They interact with each other and with other protein groups, including GTPases (COG1100), and to COGs related to extracellular matrix (Fig. 5).

## Discussion

We investigated the transcriptomic profiles of two Mediterranean sponge species upon MAMP exposure (LPS and peptidoglycan). Previous genomic information for *A*. *aerophoba* and *D*.*avara* was lacking; thus, this study provides a valuable resource with the generation of a *de novo*-assembled reference transcriptome for these species. The reference transcriptomes of *A*. *aerophoba* and *D*. *avara* contain a complex inventory of PRRs. Both species harbour hundreds of genes containing single or multiple SRCR domains, sometimes in combination with other conserved domains such as fibronectin III or immunoglobulin domains. In *D*. *avara*, 80 *bona fide* NLRs are found in the reference transcriptome. In the *A*. *aerophoba* reference transcriptome, only one gene could be identified as a *bona fide* NLR and it was constitutively expressed in all samples. However, several incomplete transcripts contained NACHT domains and could potentially add to the repertoire of expressed NLRs in this species. The NLRs represent a PRR family that is highly expanded in the *A*. *queenslandica* genome (comprising 135 genes, in contrast to 20 genes in humans)^32^; however, the reference transcriptome of the sponge *Vaceletia* sp. lacks these receptors^35^. Both *A*. *aerophoba* and *D*. *avara* constitutively express Immunoglobulin-TIR receptors, as found in other sponges^54^. In organisms with limited amenability to genetic manipulation, such as sponges, gene function is typically inferred from data from distantly-related organisms as validation of functions is challenging^27^. Consequently, the set of Poriferan-unique and species-specific traits remain misrepresented^27,57^. Nevertheless, by adopting an experimental approach, we have identified receptors and other genes that are potentially relevant to the sponge response to microbes and have narrowed the list of target genes for future research.

MAMPs (mainly LPS, but also peptidoglycan or flagellin) have been broadly used as immune activators in multiple organisms (including plants, invertebrates, and vertebrates)^8,55,58,59^. The MAMP-triggered immune pathways are considered, besides physical barriers, as the first line of the response to microbes. As filter-feeders, sponges constantly encounter diverse microbes carrying different MAMPs. To increase the chances of inducing an immune response, we chose here commercially-available MAMPs (LPS and peptidoglycan) derived from non-marine organisms. We applied them simultaneously to increase the array of transcriptionally inducible PRRs and pathways in the same treatment. For example, Zhang *et al*.^55^ showed a stronger transcriptomic response (more number of DEGs) to LPS than to peptidoglycan and fucoidan in the snail *Biomphalaria glabrata*. Similarly, Weiss *et al*.^60^ reported little overlap in the transcriptomic response of the coral *Acropora millepora* to muramyl dipeptide and poly I:C as MAMPs. The MAMP challenge is preferable over challenge with live cells when the aim is to induce the transcriptionally inducible PRRs and their activated downstream response because interference with microbial-derived effector molecules is avoided^61^. We thus consider the MAMP challenge approach meaningful for unveiling animal-microbe molecular talk, although future studies addressing other microbial challenges would help to further identify the underlying molecular mechanisms.

In invertebrates, a high diversity of PRRs and their tuned expression upon microbial stimuli has been proposed as a mechanism for specific recognition of microbes^10,19,36,62^. Here, we detected sponge species-specific signatures in the expression profiles of these PRRs upon MAMP challenge (Table 3). A SRCR domain-containing gene was up-regulated in *A*. *aerophoba* in response to MAMPs (Table 3). In *A*. *queenslandica* juveniles, more than 30 SRCR domain-containing genes with diverse architectures were differentially expressed upon exposure to microbes in aquaria experiments^38^. The implication of SRCR on microbial recognition in sponges was first evidenced by the upregulation of a SRCR-domain containing gene in symbiotic *vs* aposymbiotic (i.e., cyanobacteria-free) *Petrosia ficiformis* in the field^37^. SRCR-domain containing genes are also expanded in echinoderm genomes as well as being highly expressed in their immune cells and activated in response to microbes^63,64^. Further studies have reported the up-regulation of these receptors upon bacterial exposure in other invertebrates^65^. In *D*. *avara*, two NLRs were differentially expressed upon MAMP treatment. The complex repertoire of NLRs in *A*. *queenslandica* already hinted towards their role in microbial recognition in sponges^36^, but our findings provide the first experimental evidence of enhanced expression of poriferan NLRs in response to microbial cues. Evidence of the role of NLRs in invertebrates is scarce^66^. However, *in vitro* studies in the cnidarian *Hydra* showed that a non-conventional NLR genes (lacking the LRR domain) are differentially-expressed in response to LPS and flagellin stimulation and yield the activation of caspases in a manner that may be analogous to the mammalian inflammasome^67^.

Our study also revealed other putative immune receptors. GPCRs were differentially expressed in both *A*. *aerophoba* (up-regulated; Table 3, Supplementary Table S6) and in *D*. *avara* (down-regulated; Supplementary Table S7). The phylogenetic analysis of the *A*. *aerophoba* gene *TR165761_c4_g1* showed that it belongs to the adhesion GPCR family (Fig. 3), which is involved in adhesion and signalling. Krishnan *et al*.^68^ also classified a group of *A*. *queenslandica* adhesion GPCRs as basal of human Group I and Group II adhesion GPCRs, whereas the rest of *A*. *queenslandica* adhesion GPCRs were either sponge specific or more similar to other vertebrate GPCR families. GPCRs constitute a highly diverse receptor family in animals^25,69^, including sponges^68,70^. In vertebrates, they take part in crosstalk with microbes, by detecting microbial-derived metabolites (e.g., short-chain fatty acids) and interacting with other PRRs such as TLRs^25,71^. In invertebrates, their role in defence has been suggested for *Caenorhabditis elegans* and *Drosophila melanogaster*^24^. In addition, RNA-Seq analysis revealed that GPCR signalling played a role in the response of the sea anemone *Aiptasia* to symbiotic states and *Symbiodinium* type^72^. Thus, our results provide additional support for the conserved role of GPCRs in animal-microbe interactions. In *D*. *avara*, there is furthermore a noteworthy differential expression of several fibrinogen-domain containing genes. This domain is commonly found in the DEGs responding to microbial cues in invertebrates^55,73,74^. In addition, both species differentially expressed several genes containing immunoglobulin domains, LRR domains, DEATH domains and genes with sequence similarity to lectins (e.g. galectin). Besides their roles in cell-cell communication^75^, these domains are common in immune receptors^76^ and are involved in microbial recognition in corals^77^, snails^78^, or nematodes^79^. Moreover, a ficolin-like gene was up-regulated in the sponge *Cliona varians* when “reinfected” with *Symbiodinium* compared to the aposymbiotic tissue^80^. Therefore, GPCRs, fibrinogen-containing and lectin-like genes could add to the repertoire of genes key for immune recognition in sponges.

The response of both sponges to MAMPs involved the up-regulation of ankyrin repeat-containing genes, immunoglobulin-domain containing genes, DEATH-domain containing genes, CARD-domain containing genes and chaperones (hsp70), as well as regulation of collagen. Signalling transduction was also mediated by serine-threonine protein kinases, which were significantly down-regulated in *A*. *aerophoba* but up-regulated in *D*. *avara*. The network analyses in STRING (Figs 4, 5) show that the information available from other organisms supports the co-expression patterns reported in our study, but further studies on co-localization analysis and protein-protein interactions would be necessary to confirm these networks. These MAMP-triggered transcriptomic profiles resemble those found in other invertebrates^55,59,74,81^ and potentially mediate a high diversity of cellular responses, such as cell death^81^, phagocytosis^82^, and metabolism regulation^55^. Here, the activation of apoptosis in both species is indicated at the earliest time point (1 h). Moreover, the enhanced expression of a folate receptor (Table 4), *SRCR* and *GPCR* (Table 3) in *A*. *aerophoba* together with the differential expression of Ras family gene, *dynamin* and genes involved in cytoskeleton rearrangement (Table 4; Fig. 4) hints to the activation of a phagocytic response in this sponge species^83,84^.

We did not detect differential expression of genes encoding Immunoglobulin-TIR receptors or its adaptor protein MyD88 (myeloid differentiation primary response 88), even though both sponge species investigated here constitutively expressed Immunoglobulin-TIR domain receptors (Supplementary Tables S6, S7) and the MyD88-dependant downstream pathway (Supplementary Figs S1 and S2). In contrast, other sponge species activated *MyD88* gene in response to LPS or microbes^38,85^. In *Suberites domuncula*, *MyD88* expression was up-regulated 12 h after exposure to the same *E*.*coli*-derived LPS we used in our study^85^. However, before treatment, these sponges were kept in cultivation for a long period of time and their symbiotic bacterial load was reduced^85^, which could affect the immune reaction. Also, the combination of LPS and peptidoglycan may be a reason for the different responses reported in our study. In *A*. *queenslandica* juveniles, the up-regulation of Immunoglobulin-TIR receptors and components of the signalling pathway (including *MyD88*) was induced 2 h after exposure to bacteria^38^. The different results may be due to species-specific strategies, time-dependent responses, or the different experimental design (e.g., challenge with different microbial elicitors, different sampling points, or the use of adults *vs* juveniles).

The two species investigated here exemplify the HMA-LMA dichotomy in sponges, defined by differences in symbiont density and diversity^40,42^. Previously, Ryu *et al*. observed that SRCR, NLRs, Immunoglobulin-like, and fibronectin-3 containing genes were more abundant in the genomes of LMA than HMA sponges^34^, while Germer *et al*. found NLRs to be absent from the transcriptome of the HMA sponge *Vaceletia* sp^35^. Similarly, we observed a more abundant repertoire of NLRs in *D*. *avara* (LMA) than *A*. *aerophoba* (HMA) transcriptomes. However, comparative genome analysis would be necessary for further confirmation of this pattern between HMA and LMA sponges. In our study, both species showed certain similarities in the response to MAMPs; for example they activated apoptotic processes in the immediate response (1 h after treatment). However, the repertoire of PRR genes involved differed between species and the magnitude of the transcriptionally-regulated response (in terms of the number of DEGs) was more complex in *A*. *aerophoba* than in *D*. *avara*. In particular, further regulation of genes related with transcription and phagocytosis account for the greater transcriptomic response in *A*. *aerophoba*. These differences may point to species-specific features. For example, coral immune responses to LPS challenge and to thermal stress differ significantly depending on the species considered^61,81^. However, they may also reflect different immune strategies according to their differing HMA-LMA status. We propose that HMA sponges require a more fine-tuned regulated response to deal with potential conflicts between the signals from the MAMP stimulation and the symbiotic feedbacks from their highly dense microbial community. In line with the Danger model of immunity^18^, we further hypothesize that the host danger signals released upon apoptosis subsequently trigger an enhanced immune response and phagocytic activity. This hypothesis is supported in *A*. *aerophoba* by an increased expression of apoptosis genes after 1 h and of phagocytosis-related signalling pathways after 3 h of MAMP challenge. Further studies including more HMA-LMA species are on-going to elucidate whether the HMA-LMA status contributes to the variation in immune responses to microorganisms among sponge species.

## Conclusions

The characterization of the innate immune response through experiments and functional studies remains limited to few animal groups and was previously lacking in the phylum Porifera. We exposed two Mediterranean sponge species to MAMPs (LPS and peptidoglycan) and described, to our knowledge for the first time, the response of the sponges to immune stimuli by RNA-Seq. The sponges responded by increased expression of a subset of relevant receptors (i.e., NLRs in *D*. *avara*, SRCR and GPCRs in *A*. *aerophoba*) and the transduction of signals by kinase cascades that likely yield apoptosis and regulation of metabolic processes. In addition, the magnitude of the transcriptomic response was higher in *A*. *aerophoba* and this was related to the regulation of additional processes such as phagocytosis. The differences between species in the subset of regulated receptors and pathways when exposed to MAMPs may relate to their different symbiont load (HMA/LMA status). We propose that the presence of a highly dense symbiotic community in *A*. *aerophoba* influences the signalling feedbacks and determines the more complex transcriptomic response upon MAMP challenge in this species. Our findings address a prominent gap in marine sponge research by providing novel information on the repertoire of genes involved in immune recognition and signalling in this ancient animal phylum.

## Electronic supplementary material

Supplementary information

Table S1

Table S2

Table S3

Table S4

Table S6

Table S7

## Data Availability

Raw reads with the corresponding metadata and gene quantification matrices generated during the current study are available in the ArrayExpress database at EMBL-EBI archive (www.ebi.ac.uk/arrayexpress) under accession number E-MTAB-6757. D*e novo* reference transcriptomes and their full annotation are available from the corresponding author upon request. Further processed data are included in this article and its Supplementary Information files.
