# Supplementary material for: Differential expression of immune receptors in two marine sponges upon exposure to microbial-associated molecular patterns
Source: Sci Rep. 2018 Oct 31;8:16081. doi: 10.1038/s41598-018-34330-w (PMC6208332; doi:10.1038/s41598-018-34330-w)
Supplement: Supplementary file 1 — Supplementary information [file 41598_2018_34330_MOESM1_ESM.pdf]

Supplementary Information

Differential expression of immune receptors in two marine sponges upon exposure to microbial-associated molecular patterns

Lucía Pita, Marc P. Hoeppner, Marta Ribes and Ute Hentschel.

lpita@geomar.de

Supplementary Tables

Table S1. Annotated genes in *A. aerophoba* reference transcriptome containing conserved domains that characterise PRRs: TIR domain, NACHT domain, and SRCR domain.

Table S2. Annotated genes in *D. avara* reference transcriptome containing conserved domains that characterise PRRs: TIR domain, NACHT domain, and SRCR domain.

Table S3. Differential gene expression analysis comparing *A. aerophoba* samples in MAMP vs control treatment within each time point by edgeR (genes with FDR p-value < 0.05). logFC: log (fold change), negative values denote down-regulation, positive values denote up-regulation in the MAMP treatment compared to control. Log CPM: log (counts per million). FDR: false discovery rate-corrected p-value.

Table S4. Differential gene expression analysis comparing *D. avara* samples in MAMP vs control treatment within each time point by edgeR (genes with FDR p-value < 0.05). logFC: log (fold change), negative values denote down-regulation, positive values denote up-regulation in the MAMP treatment compared to control. Log CPM: log (counts per million). FDR: false discovery rate-corrected p-value.

Table S5. Differentially expressed genes (FDR p-value < 0.005) *in A. aerophoba* that are involved in metabolism, gene expression and other functions.

Table S6. Full annotation of differentially expressed genes (FDR p-value < 0.05) in *A. aerophoba*.

Table S7. Full annotation of differentially expressed genes (FDR p-value < 0.05) in *D. avara*.

Table S5.

Gene Description	Gene IDs	Time	LogFC	FDR
**Metabolism**				
Long-chain-fatty-acid-CoA ligase	TR142631_c0_g2	3h	4.7	**0.003**
	TR142631_c0_g9	3h	5.2	**0.003**
	TR142631_c0_g18	3h	6.7	**2.6 e-5**
	TR142631_c0_g21	3h	6.1	**2.8 e-6**
	TR125500_c0_g7	3h	-5.2	**4.6 e-5**
PP2Cc	TR153708_c0_g1	3h	3.9	**2.0 e-4**
3-oxoacid CoA-transferase 2A	TR66545_c0_g1	1h	-8.2	**6.4 e-4**
Acyl-CoA binding domain containing gene	TR150854_c0_g3	5h	-9.3	**0.002**
Aldehyde dehydrogenase	TR156897_c3_g16	5h	9.5	**2.5 e-5**
Cytochrome c oxidase assembly factor 3	TR131371_c0_g3	1h	3.5	**4.9 e-3**
Ubiquinone	TR164188_c2_g2	3h	7.3	**0.001**
Polysaccharide deacetylase	TR165674_c0_g1	3h	4.4	**8.8 e-4**
Phosphoglycerate mutase	TR166027_c9_g4	1h;<br>3h	4.8;<br>5.5	**1.5 e-4;**<br>**4.9 e-4**
	TR166027_c9_g6	1h	-5.6	**7.1 e-5**
Transketolase	TR72158_c0_g1	3h	3.9	**2.7 e-4**
	TR72158_c0_g3<br>TR72158_c0_g4	1h;<br>3h;<br>5h	-3.9;<br>-4.9;<br>-4.6	**0.004;**<br>**1.8 e-4;**<br>**2.9 e-4**
	TR72158_c0_g5	3h	3.9	**4.2 e-4**
Transglutaminase	TR167657_c5_g1	3h	7.4	**0.003**
Adenylosuccinate ligase	TR301111_c0_g1	5h	-8.9	**5.2 e-5**
Aminotransferase	TR164899_c5_g3	3h	-5.7	**8.2 e-5**
Potassium channel tetramerization containing domain	TR139584_c0_g2	5h	-7.9	**0.001**
Ribosomal proteins	TR138971_c2_g2	5h	-8.8	**2.6 e-5**
	TR154264_c1_g2	1h;<br>3h	7.7;<br>7.5	**0.001;**<br>**0.001**
	TR154264_c1_g5	1h;<br>3h;<br>5h	-8.2;<br>-8.1;<br>-8.7	**1.1 e-4;**<br>**5.4 e-5;**<br>**1.1 e-4**
	TR154264_c3_g2	3h	3.4	**0.003**

Table S5. ***(cont)***

Gene Description	Gene IDs	Time	LogFC	FDR
**DNA binding/regulation of transcription**				
Pentatricopeptide repeat-containing gene	TR119847_c0_g1	1h;<br>3h	8.1;<br>7.8	**1.1 e-4;**<br>**9.7 e-4**
Endonucleases	TR150148_c0_g1	5h	8.0	**0.003**
	TR173868_c3_g2	3h	5.8	**0.004**
	TR170028_c2_g3	1h	7.7	**4.6 e-3**
	TR171176_c2_g2	3h	8.3	**1.2 e-8**
	TR173961_c1_g1	3h	8.0	**2.7 e-4**
	TR167214_c1_g2	3h	-5.7	**0.004**
	TR172196_c2_g5	1h	-10.0	**8.5 e-4**
Transposases	TR157594_c1_g10	5h	6.0	**1.7 e-4**
	TR157594_c1_g16	5h	5.6	**1.4 e-4**
	TR163992_c2_g3	3h	-10.1	**5.2 e-9**
Polymerase	TR171723_c1_g28	1h;<br>3h;<br>5h	7.3;<br>6.3;<br>8.9	**0.002;**<br>**0.003;**<br>**4.5 e-3**
Transcription factors	TR174280_c6_g4	1h;<br>3h;<br>5h	8.6;<br>7.9;<br>8.2	**1.0 e-4;**<br>**9.4 e-5;**<br>**0.002**
	TR163347_c3_g4	3h	8.3	**0.002**
Zinc knuckle and retroviral aspartyl protease domain-containing gene	TR168355_c5_g4	3h	7.7	**4.2 e-3**
Zinc finger protein like	TR170073_c2_g3	3h	-8.2	**9.2 e-5**
Ependymin-related	TR174135_c4_g21	5h	-7.6	**6.8 e-9**

Gene description is based on domain annotation and/or blast results. Supplementary Information provides full information on annotation (including e-values) (Supplementary TableS6) and full DGE results (here we provide rounded log2 fold change and FDR p-values, full values are reported in Supplementary TableS3). Log FC: log_2 (fold change). Positive values of Log FC denote up-regulated genes and are coloured in orange; negative values of log FC denote down-regulated genes and are coloured in blue. FDR: false discovery rate-corrected p-value.

Supplementary Figures

Figure S1. Components of the Toll-like receptor signalling pathway identified in the reference transcriptome of *A. aerophoba* by KEGG annotation (in red). KEGG: Kyoto Encyclopaedia of Genes and Genomes (Kanehisa et al. 2017). Publication permission granted by Kanehisa laboratories (Ref. 180230).

Figure S2. Components of the Toll-like receptor signalling pathway identified in the reference transcriptome of *D. avara* by KEGG annotation (in red). KEGG: Kyoto Encyclopaedia of Genes and Genomes (Kanehisa et al. 2017). Publication permission granted by Kanehisa laboratories (Ref. 180230).

Figure S3. Number of differentially-expressed genes per time point (FDR p-value < 0.005) for each species as obtained by DESeq2 analysis.

References

Kanehisa, M., Furumichi, M., Tanabe, M., Sato, Y., and Morishima, K. (2017). KEGG: New perspectives on genomes, pathways, diseases and drugs. *Nucleic Acids Res*. **45**, D353–D361. doi:10.1093/nar/gkw1092.

Figure S1-KEGG map visualization of the Toll-like receptor signalling pathway in the reference transcriptome of *A. aerophoba*

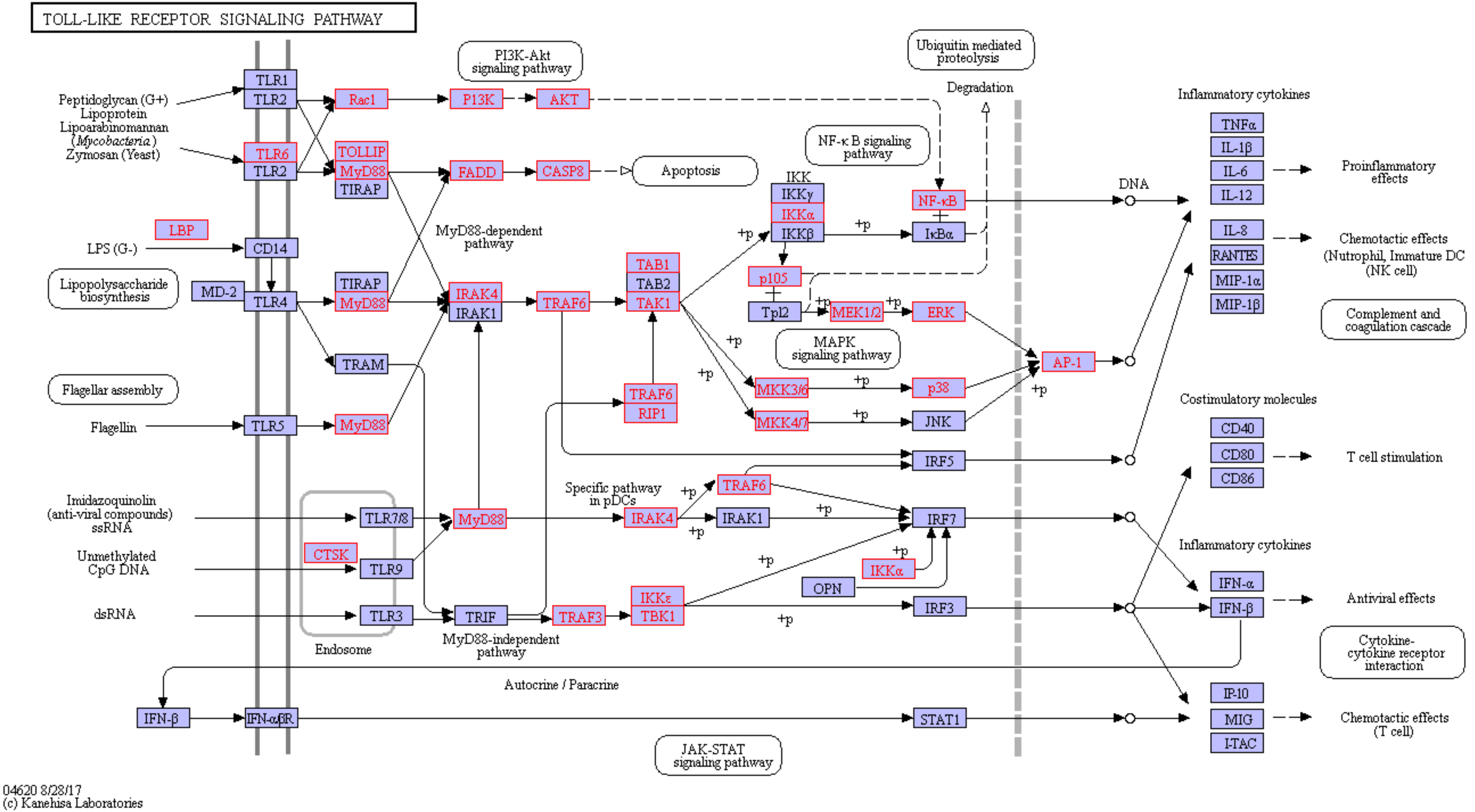

Figure S2- KEGG map visualization of the Toll-like receptor pathway in the reference transcriptome of *D. avara*

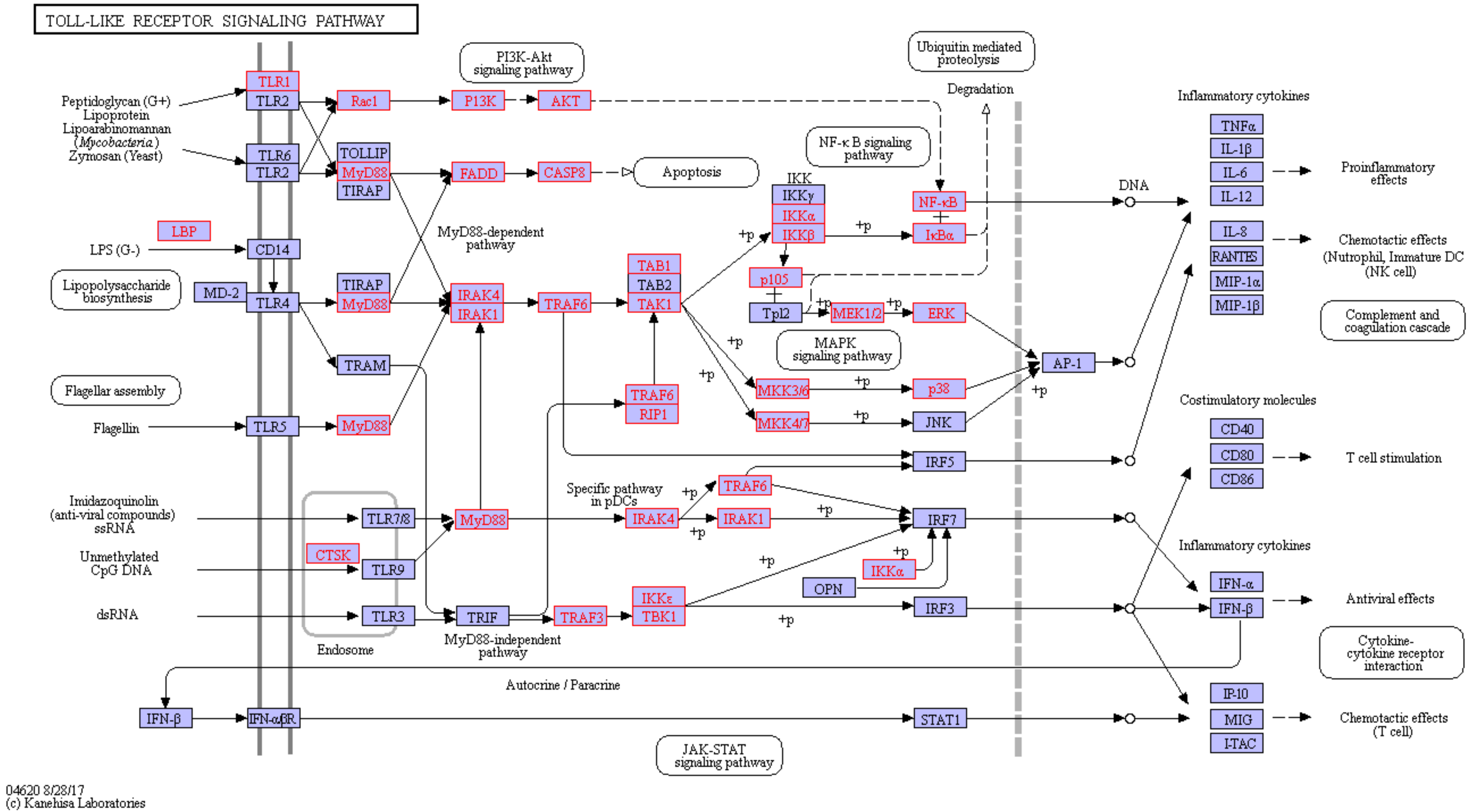

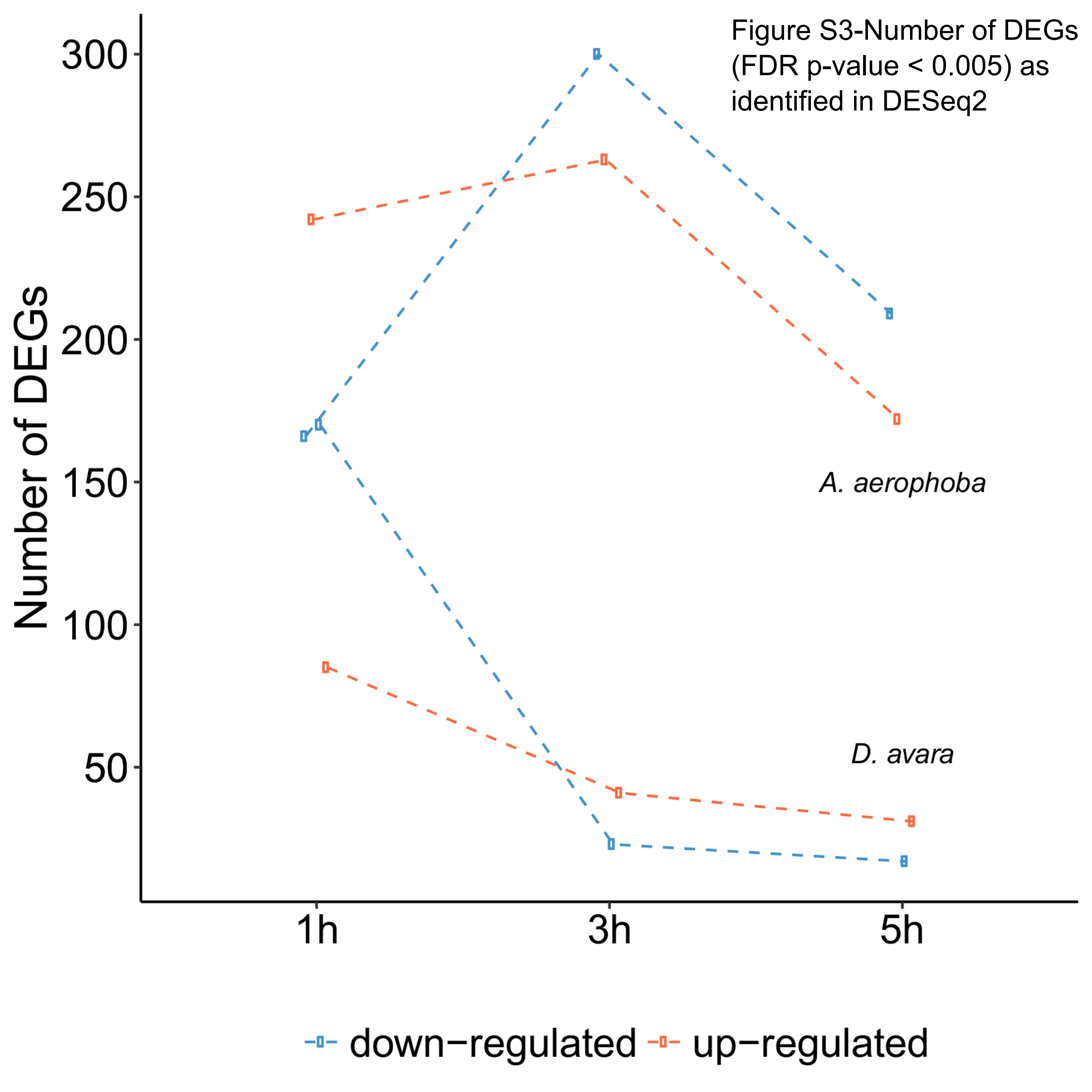
Figure S3-Number of DEGs (FDR p-value < 0.005) as identified in DESeq2
Number of DEGs
300
250
200
150
100
50
1h
3h
5h
A. aerophoba
D. avara
down−regulated
up−regulated
